# Daily Automated Prediction of Delirium Risk in Hospitalized Patients: Model Development and Validation

**DOI:** 10.2196/60442

**Published:** 2025-04-18

**Authors:** Kendrick Matthew Shaw, Yu-Ping Shao, Manohar Ghanta, Valdery Moura Junior, Eyal Y Kimchi, Timothy T Houle, Oluwaseun Akeju, Michael Brandon Westover

**Affiliations:** 1 Department of Anesthesia, Pain, and Critical care Medicine Massachusetts General Hospital Boston, MA United States; 2 Harvard Medical School Boston, MA United States; 3 Department of Neurology Massachusetts General Hospital Boston, MA United States; 4 Department of Neurology Beth Israel Deaconess Medical Center Boston, MA United States; 5 Ken & Ruth Davee Department of Neurology Feinberg School of Medicine Northwestern University Chicago, IL United States

**Keywords:** delirium, prediction model, machine learning, boosted trees, model development, validation, AI, artificial intelligence, screening, prevention, develop, logistic regression, vitals, vital signs, gender, age, prevent

## Abstract

**Background:**

Delirium is common in hospitalized patients and is correlated with increased morbidity and mortality. Despite this, delirium is underdiagnosed, and many institutions do not have sufficient resources to consistently apply effective screening and prevention.

**Objective:**

This study aims to develop a machine learning algorithm to identify patients at the highest risk of delirium in the hospital each day in an automated fashion based on data available in the electronic medical record, reducing the barrier to large-scale delirium screening.

**Methods:**

We developed and compared multiple machine learning models on a retrospective dataset of all hospitalized adult patients with recorded Confusion Assessment Method (CAM) screens at a major academic medical center from April 2, 2016, to January 16, 2019, comprising 23,006 patients. The patient’s age, gender, and all available laboratory values, vital signs, prior CAM screens, and medication administrations were used as potential predictors. Four machine learning approaches were investigated: logistic regression with L1-regularization, multilayer perceptrons, random forests, and boosted trees. Model development used 80% of the patients; the remaining 20% was reserved for testing the final models. Laboratory values, vital signs, medications, gender, and age were used to predict a positive CAM screen in the next 24 hours.

**Results:**

The boosted tree model achieved the greatest predictive power, with an area under the receiver operator characteristic curve (AUROC) of 0.92 (95% CI 0.913-9.22), followed by the random forest (AUROC 0.91, 95% CI 0.909-0.918), multilayer perceptron (AUROC 0.86, 95% CI 0.850-0.861), and logistic regression (AUROC 0.85, 95% CI 0.841-0.852). These AUROCs decreased to 0.78-0.82 and 0.74-0.80 when limited to patients who currently do not or never have had delirium, respectively.

**Conclusions:**

A boosted tree machine learning model was able to identify hospitalized patients at elevated risk for delirium in the next 24 hours. This may allow for automated delirium risk screening and more precise targeting of proven and investigational interventions to prevent delirium.

## Introduction

Delirium is a common condition in hospitalized patients and has been recognized as an independent risk factor for poor clinical outcomes, including mortality, institutionalization, and cognitive impairment following hospital discharge [1-3]. The US annual national costs attributable to delirium have been estimated to be as high as US $152 billion, rivaling costs attributable to diabetes and falls [4]. As a result, a basic assessment for delirium is recommended for all hospitalized patients aged 65 years or older [5], and formal screening for delirium is recommended for critically ill patients [6].

Despite these recommendations, delirium frequently remains undiagnosed [7]. An automated delirium prediction tool could help address this, by alerting clinicians to at-risk patients so that they could be more carefully assessed for delirium. Such screening tools could also help focus interventions aimed at the prevention of delirium (eg, components of the hospital elder life program [8]) and provide an enriched patient sample for future delirium prevention studies.

In particular, we intend to use an automated tool to identify hospitalized patients at our institution who are at high risk of delirium in the next 24 hours. These patients will then be visited by a member of a delirium service for further evaluation and identification of interventions that may reduce the patient’s risk of delirium. For this purpose, near-term risk (24-h risk) is more useful than the risk of delirium at some point during this hospitalization, and any history of prior or current delirium is relevant to identifying the patients at risk of ongoing delirium who should be seen (as reducing the duration of ongoing delirium is still likely to benefit the patient).

Although multiple prior delirium prediction tools have been described [9-12] (for a recent systematic review, see [13]) most have properties that have limited their use as a tool to be applied daily to every patient in the hospital. Most prediction tools are designed to allow a risk score to be easily calculated by a clinician by hand, limiting the model’s performance compared to larger models with more features and favoring features that are easy for a human to produce over those easily extracted from the medical record. In addition, most prior models were developed using datasets of only a few hundred to a few thousand patients, limiting the complexity of the models that could be developed without overfitting.

To address these limitations, we have developed a model that can provide automated delirium screening based on data readily available from the electronic health record, emphasizing predictive power over ease of manual computation or ease of interpretation. Because current and prior delirium are known risk factors for future delirium, we also explore the performance of the model in patients without these risk factors. This tool achieves state-of-the-art accuracy for delirium prediction in this automated setting and maintains good performance even when restricted to patients without current or prior delirium.

## Methods

### Ethical Considerations

This study was reviewed and approved by the Mass General Brigham institutional review board (approval 2013P001024). The institutional review board determined that informed consent was not required for this retrospective study. This study adheres to the applicable TRIPOD (Transparent Reporting of a multivariable prediction model for Individual Prognosis Or Diagnosis) guidelines.

### Study Cohort

Data were obtained for all patients who received any variation of a Confusion Assessment Method (CAM) screen [14] (eg, the CAM-ICU) [15]) in our hospital between April 2, 2016, and January 16, 2019, for a total of 23,006 patients. No specific exclusion criteria were used, as we wished the results to be applicable to the typical population of the hospital. Approximately 20% (n=4511) of patients were randomly selected and set aside for the final evaluation of the model (the “test dataset”); we remained blind to this dataset until after all model choices and parameters had been fixed in preparation for publication. The remaining 80% of patients (the “training dataset”) were used for model selection, model training, and hyperparameter tuning.

### Model Development Overview

We provide an overview of the model development here; additional details can be found in Multimedia Appendix 1.

For the outcome to be predicted, we used the presence of at least one positive CAM screen within a given day where CAM screens were performed. The CAM screen is a validated and widely used tool for assessing delirium where an observer assesses for a change in cognition with an acute onset and fluctuating course involving inattention and either disorganized thinking or an altered level of consciousness. [14] For each patient, we first identified all 24-hour intervals from 5 AM to 5 AM during which at least one CAM screen or CAM screen variation had been performed. For each such interval, the model was required to predict whether at least one CAM screen variant would be positive during that interval (vs all negative CAM screens).

As model inputs, we used the patient’s age, gender, and all prior recorded vital signs, laboratory values, medications, and prior CAM assessments present in the medical record at 5 AM before the 24 hours in which delirium was to be predicted. Categorical values were converted to integers (eg, “1” for “Positive,” “0” for “Negative”). These data were reduced to summary statistics for each measurement (eg, minimum, maximum, and mean systolic blood pressure in the past 24 h), which were used to form fixed-length feature vectors for each prediction interval. These feature vectors were then normalized by subtracting the median and dividing by the interquartile interval, with both the median and the quartiles estimated by the P2 algorithm [16]. Because the P2 algorithm provides only an approximation of the quantiles, the resulting values were generally not exact integers even for categorical values (eg, a binary measure that was mostly negative would have an estimated median that was slightly above 0). Features that were missing in more than 95% of the patients were discarded. The remaining missing values were imputed to be the (P2-estimated) median value for the feature. To provide a consistent basis of comparison, we used these imputed values for all the models, including those (such as boosted trees) that do not strictly require imputation. Because of the sparsity of these features, this implies that most features for a patient will be at the median (imputed) value, especially for the first patient snapshot. Of note, however, because the P2-estimated median is a unique value that is otherwise infrequent in the data, nonlinear models can use this as a marker for missing data (and this missingness itself may have predictive value).

In effect, we are asking the model to produce a single prediction each morning of whether the patient will have delirium later that day using all of the information available that morning (including prior CAM screens). In this study, we do not train or test the performance of the model when used on a rolling basis throughout the day (eg, to generate a new prediction at 4 PM incorporating data from 3 PM that day).

The XGBoost library [17] was used to fit boosted tree models [18] for the cleaned and normalized training datasets. For comparison, random forest models [19] and logistic regression models using L1 regularization [20] were also fit to the data using scikit-learn [21]. In addition, a deep neural network model was developed using TensorFlow (Google) [22]. The final network had a 32-node rectified linear unit [23] input layer, 2 hidden layers of 16 and 8 rectified linear unit nodes, respectively, and an output layer with a single sigmoidal node. All layers were fully connected, and a 50% dropout [24] was used between layers. For the logistic regression and random forest models, Platt scaling was used to improve the calibration of the model. We used 10-fold cross-validation [25] for hyperparameter tuning to minimize overfitting. Hyperparameters (such as lambda for L1 regularization) were tuned using a grid search.

All development was done using the Ubuntu 18.04 distribution of Gnu-Linux. Data processing and analysis were performed using the Python [26] and Julia [27] programming languages. The code used to generate the models and figures is publicly available [28]. The datasets used to develop and test the model contain personally identifiable health information, and thus, are not publicly available; the authors can be contacted for more information.

### Statistical Analysis

We provide an overview of the statistical analysis here; additional details can be found in Multimedia Appendix 2. The receiver operator characteristic curve and the area underneath the receiver operating characteristic curve (AUROC) were used to evaluate the performance of each model. To capture the effects of population prevalence on performance, we also used precision-recall curves and the area under the precision-recall curves (AUPRC). Calibration curves were used to qualitatively evaluate model calibration, and the expected calibration error (ECE) and maximum calibration error (MCE) were used to quantify the degree of calibration. CIs were calculated in Python using bootstrapping with 1000 rounds, and resampling by prediction day. To interpret the final behavior of the models, we used Shapley Additive Explanations (SHAP) value estimation methods as described by Lundberg et al [29,30].

The final models were trained on the full training dataset (80% of patients). Once the final models were trained, the test dataset was unblinded and the model performance was measured on the test dataset. The performance of the cross-validated version of the models on the training dataset was similar to the performance of the final models on the test dataset and is not reported here.

Although current and prior delirium are useful predictors for our intended use of the model, these are well-known risk factors for delirium and it is thus useful to explore how the model performs on patients without these risk factors. To test the model performance of these populations with a lower initial probability of delirium, versions of the final models were trained and tested on only snapshots of patients who did not have delirium (ie, no positive CAM screens in the past 24 h) or never had delirium (no prior positive CAM screens). Conceptually, all patients start in the “never delirious” state, and then potentially transition to a “currently delirious” state and then potentially between this state and a “previously but not currently delirious” state. For a summary of the assignment of patient snapshots to these groups, please see Multimedia Appendix 3.

## Results

Of the 20,006 patients in the dataset, 4583 (19.9%) patients had at least one positive CAM screen (Table 1). The average age of the patients was 65 (SD 17) years and slightly higher in patients with a positive CAM screen (mean 70, SD 16 years). Approximately, 54% (n=12,502) of patients were male and 46% (n=10,500) of patients were female; no other genders were recorded in this dataset. The fraction of male patients was slightly higher (2614/4582, 57%) in patients with a positive CAM screen. An average of 12.6 (SD 17.9) CAM screens were recorded per patient, with more (mean 24.8, SD 29) recorded for patients with a positive CAM screen than for patients with no positive CAM screens (mean 9.5, SD 12). An average of 8.3% (SD 22%) of CAM screens per patient were positive, which rose to an average of 42% (SD 33%) of CAM screens per patient that were positive in patients who had at least one positive CAM screen. CAM screens were performed on an average of 8.1 (SD 11) days per patient, of which 8.1% (SD 21%) of days with CAM screens had at least one positive CAM screen.

**Figure 1:**
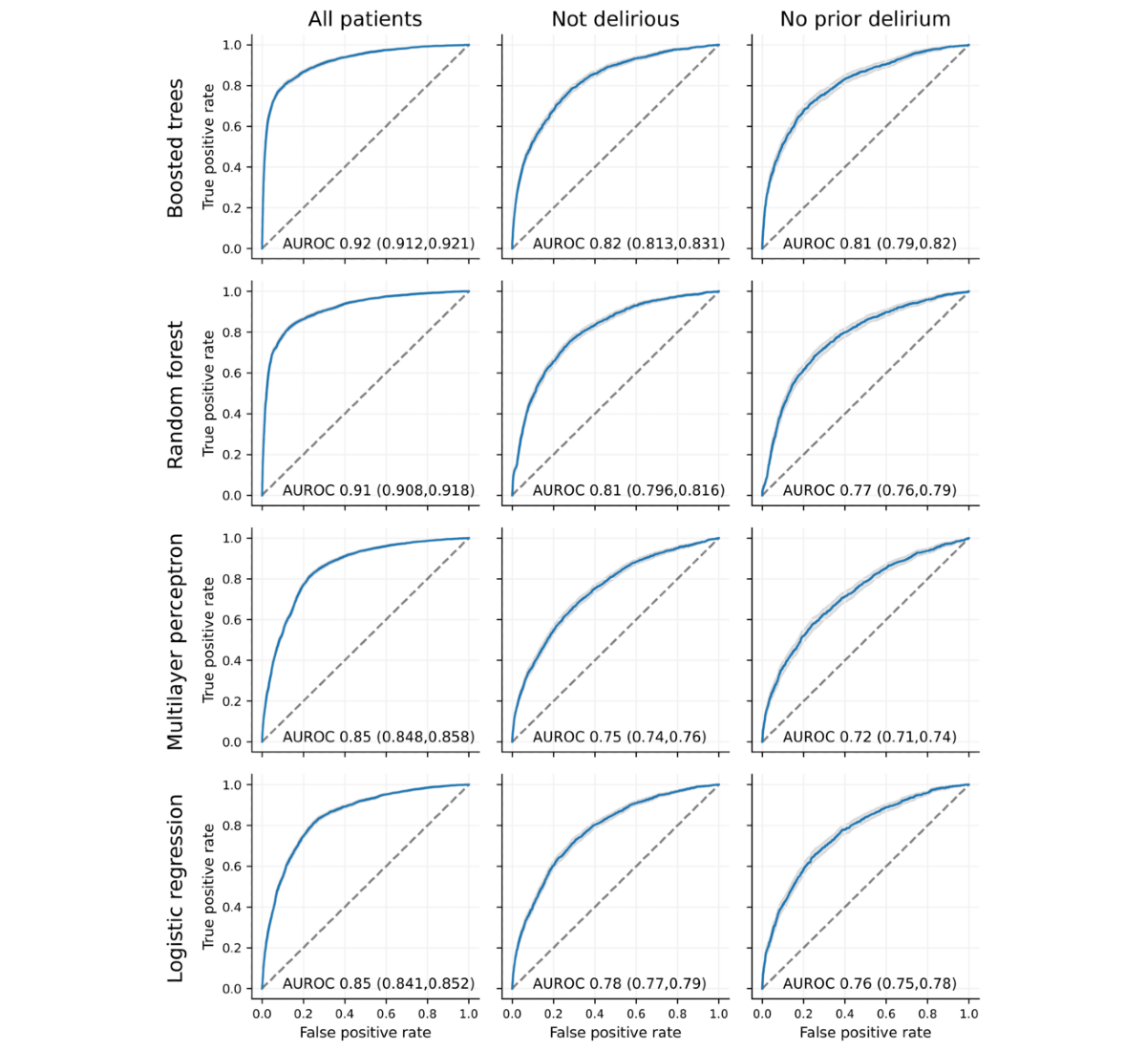
Receiver operator characteristic curves for different model types (rows) and patient subsets (columns) showing the true positive rate (ie, recall) as a function of the false positive rate. The thin light gray region around the line shows the bootstrap 95% CI. AUROC: area under the receiver operator characteristic curve.

All models provided significant predictive power for delirium (a positive CAM screen in the next 24 h) when applied to all hospitalized patients in the dataset (Figure 1). The boosted tree model had the highest AUROC (0.92, 95% CI 0.913-9.22). This was followed by the random forest model, the multilayer perceptron, and the logistic regression model with L1-regularization (AUROC 0.85, 95% CI 0.841-0.852).

**Table 1:** Patient demographics.

Measure	All patients (n=23,006)	Training set (n=18,495)	Testing set (n=4511)	One or more positive CAM^a^ (n=4583)	All CAM negative (n=18,423)
Age (years), mean (SD)	65.5 (17.3)	65.5 (17.3)	65.5 (17.2)	69.8 (16.1)	64.4 (17.4)
Male, n (%)	12,502 (54.3)	10,037 (54.3)	2465 (54.6)	2614 (57)	9888 (53.7)
Female, n (%)	10,500 (45.6)	8455 (45.7)	2045 (45.3)	1968 (42.9)	8532 (46.3)
Patients with at least one positive CAM screen, n (%)	4583 (19.9)	3656 (19.8)	927 (20.5)	4583 (100)	0 (0)
CAM evaluations per patient, mean (SD)	12.6 (17.9)	12.5 (18.0)	12.9 (17.6)	24.8 (29.0)	9.5 (12.0)
Percent of positive CAM screens per patient, mean (SD)	8.3 (22)	8.3 (22)	8.3 (22)	42 (33)	0 (0)
CAM evaluation days per patient, mean (SD)	8.1 (11.1)	8.1 (11.3)	8.3 (10.5)	15.6 (18.0)	6.2 (7.6)
Percent positive CAM days, mean (SD)	8.1 (21)	8.1 (21)	8.2 (21)	40.8 (31)	0 (0)

^a^CAM: Confusion Assessment Method.

The models were then retrained and evaluated with patients who did not currently have delirium (most recent CAM screen was negative) and with patients who had no history of delirium (no prior positive CAM screens). Although the models did not perform as well on these more difficult subsets, they still provided good predictive power. The boosted tree model declined from an AUROC of 0.92 to an AUROC of 0.82 (95% CI 0.815-0.834) and 0.80 (95% CI 0.79-0.81) when limited to patients who did not currently have delirium and patients with no prior delirium, respectively. The other models showed a similar decrement, with the AUROC decreasing to 0.77-0.81 and 0.74-0.77 when limited to patients who do not currently have delirium and those who never have had delirium, respectively. The boosted tree model outperformed the other models in all three patient groups.

**Figure 2:**
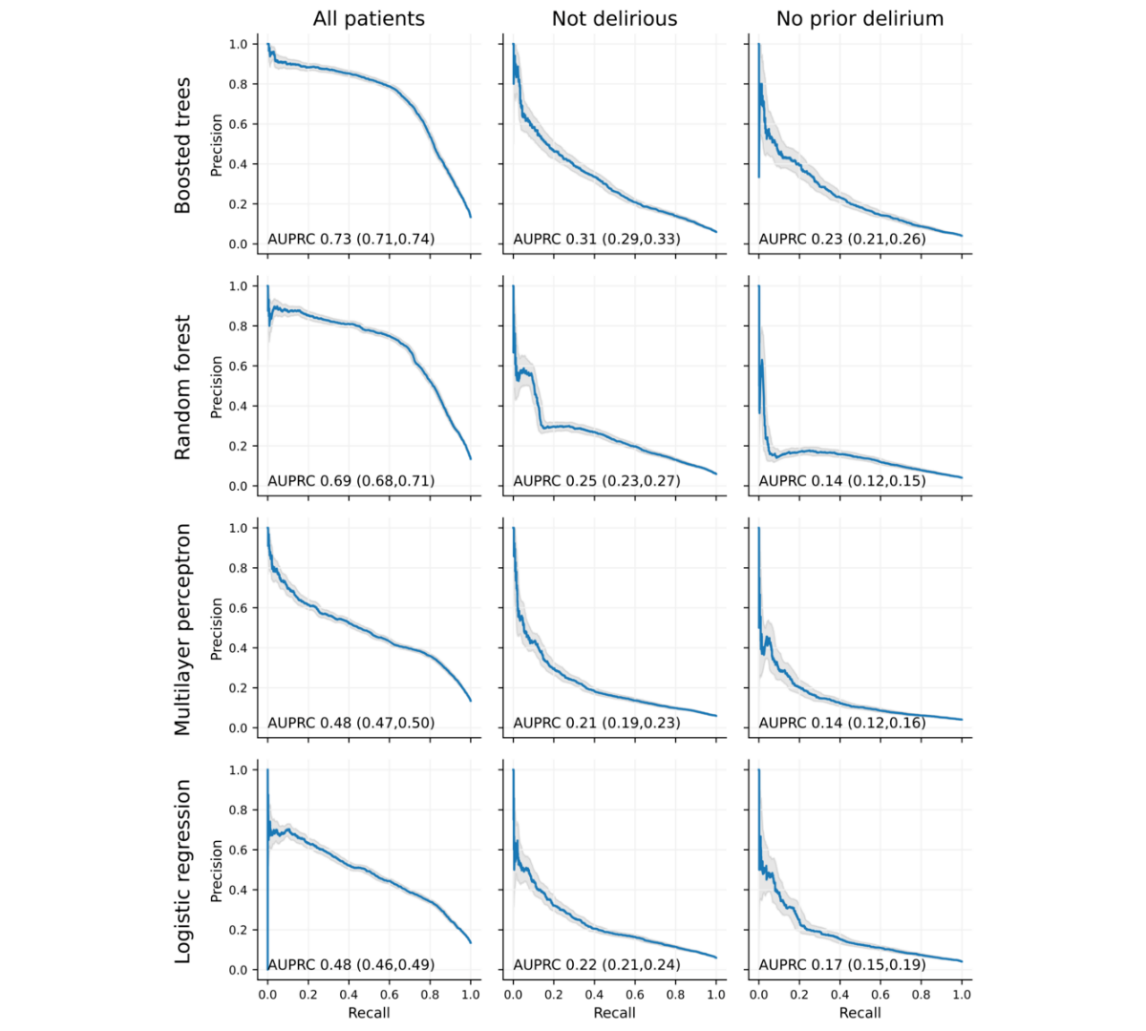
Precision-recall curves for different model types (rows) and patient subsets (columns) showing precision (ie, positive predictive value) as a function of recall (ie, true positive rate). The gray region indicates the bootstrap 95% CI.

The models significantly varied in their ability to maintain a high positive predictive value as sensitivity was increased (Figure 2). The boosted tree model performed well (AUPRC 0.73, 95% CI 0.72-0.75), its performance declining significantly (AUPRC 0.32, 95% CI 0.30-0.34) for patients who do not currently have delirium and (0.22, 95% CI 0.20-0.25) those with no prior delirium. The incidence of delirium in all patients was 13%, those with no current delirium 6%, and those who never have had delirium 4%; thus, the decrement in AUPRC appears to be largely driven by the decreased incidence in these subgroups. While the random forest model performs relatively (AUPRC 0.70, 95% CI 0.68-0.71), it also experiences significant decrements in performance with patients who do not currently have delirium (AUPRC 0.25) or have no history of delirium (AUPRC 0.14). The multilayer perceptron models perform somewhat worse than the tree-based models, with AUPRCs of 0.50, 0.23, and 0.15 in all patients, those who do not currently have delirium, and those with no prior delirium groups. Logistic regression performed similarly to the multilayer perceptron with AUPRCs of 0.48, 0.22, and 0.17.

**Figure 3:**
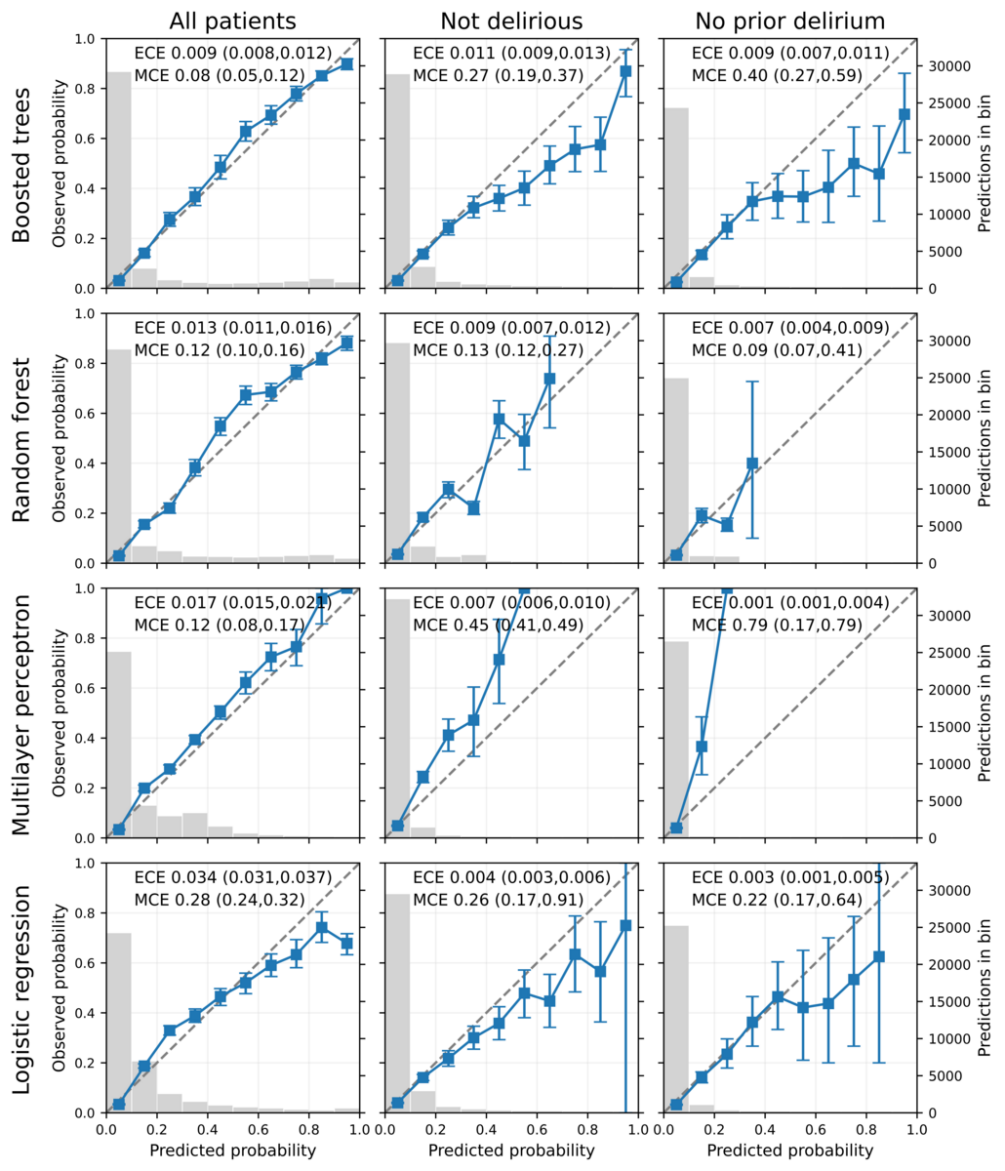
Reliability diagrams for different model types (rows) and patient subsets (columns) showing the actual fraction of patient snapshots with delirium for groups with a given predicted risk of delirium (blue squares, left y-axis). Error bars show the bootstrap 95% CI. The gray bars in the background show the number of patient snapshots in each predicted probability bin (y-axis on the right). ECE and MCE are with a 95% CI. ECE: expected calibration error; MCE: maximum calibration error.

We next investigated the calibration of the prediction models. All the models were well calibrated (ECE≤0.02; MCE≤0.11) when applied to all hospitalized patients (Figure 3), with the exception of the logistic regression model which overestimated the risk of delirium in the highest-scored group (ECE 0.03; MCE 0.28). The boosted tree model and the random forest model both identified a larger number of high-risk patients while still maintaining good calibration in this higher-risk group. When restricted to patients who do not currently have delirium or to patients with no prior delirium, all models classified very few patients as high risk (consistent with the earlier precision-recall curves), and the random forest did not assign higher probabilities to any patients in these subgroups.

**Figure 4:**
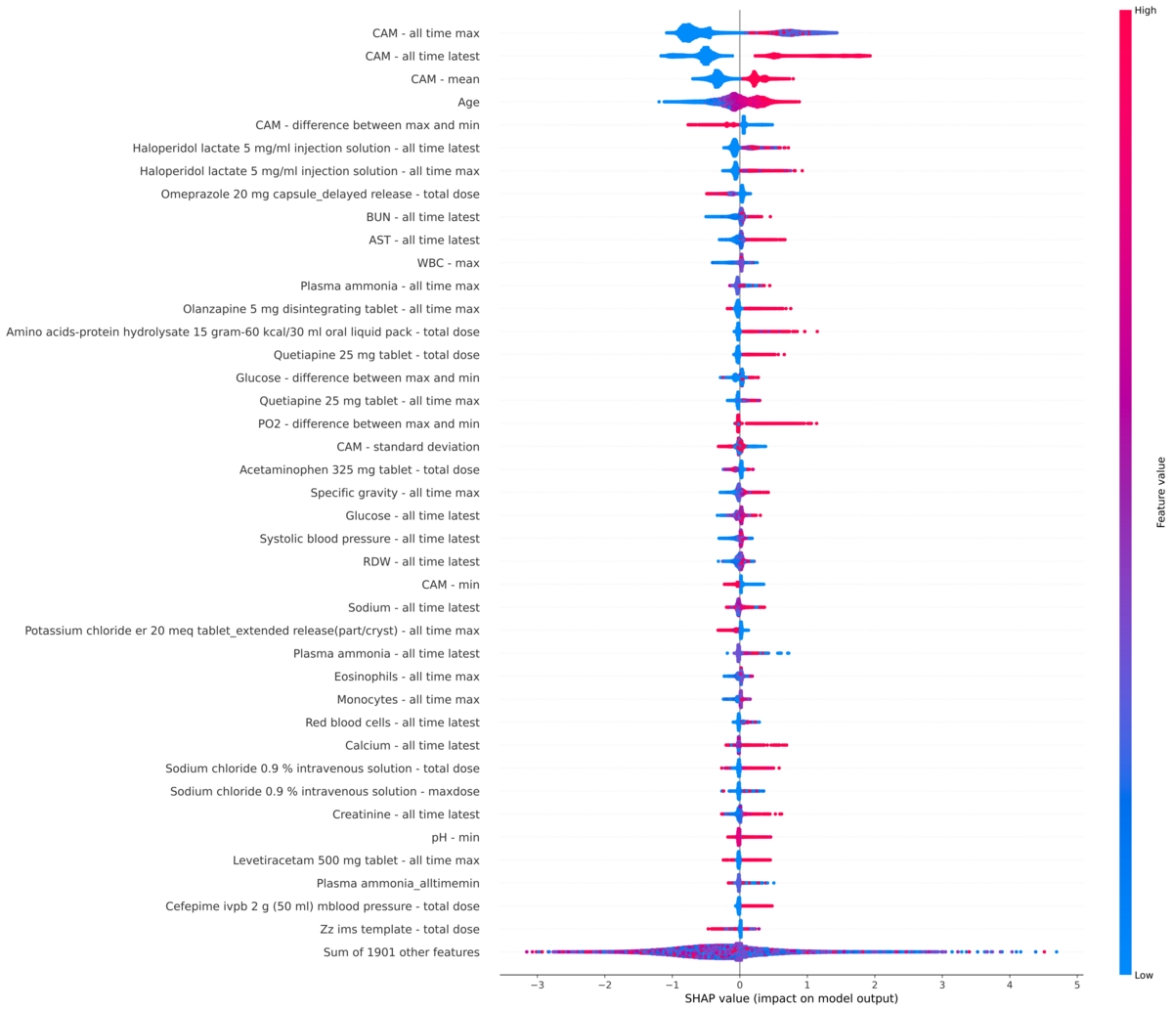
SHAP beeswarm plots [28,29] of the 40 features with the highest SHAP magnitude for patients in the holdout dataset. Each dot shows a single prediction for a patient, the color of the dot indicates how high (red) or low (blue) the feature was for this patient, and the horizontal position of the dot indicates the relative effect of this feature on the predicted risk for the given patient.

We finally turn to an examination of the features influencing the predictions of the most successful model (the boosted tree model). Ordering the features by average SHAP magnitude (Figure 4 [29,30]), we first note that the range of SHAP values for any of the top 40 features is smaller than the range of SHAP values for the sum of the remaining 1901 features; thus, the predictions of the model across cannot easily be simplified to a small number of driving features that are the same for all patients. The features with the highest average SHAP magnitude appear to fall into several known risk factors for delirium. Current and prior delirium is a known predictor of future delirium, and 6 of the top 40 features relate to the prior CAM screen (including 4 of the top 5 features). Of note, the model considers a patient with no prior CAM screens to be at higher risk than a patient with prior negative CAM screens, and this feature remains important even for the “no prior delirium” case (not shown). Antipsychotic administration is also as expected a risk-predicting feature, as it is often used to convert hyperactive delirium to hypoactive delirium. The majority of the top 40 risk features fall into other categories, however, which include known risk factors such as age, liver failure (eg, aspartate transferase levels, ammonia levels, and hepatitis C virus levels), infection (eg, white blood cells, monocytes, and cefepime [which is also neurotoxic]), and malnutrition or frailty (amino acid supplementation, albumin levels).

Of note, many of the top features had missing values in the majority of patients (Multimedia Appendix 4). These often-included values such as neutrophil counts from cerebrospinal fluid, where the presence of the measurement itself suggests a high risk of delirium. This is again consistent with what is seen with the SHAP plot, where the models are able to use a constellation of low-frequency features of each patient to determine delirium risk (eg, laboratories reflecting concern for meningitis) rather than being limited to a few broad risk factors such as age.

**Figure 5:**
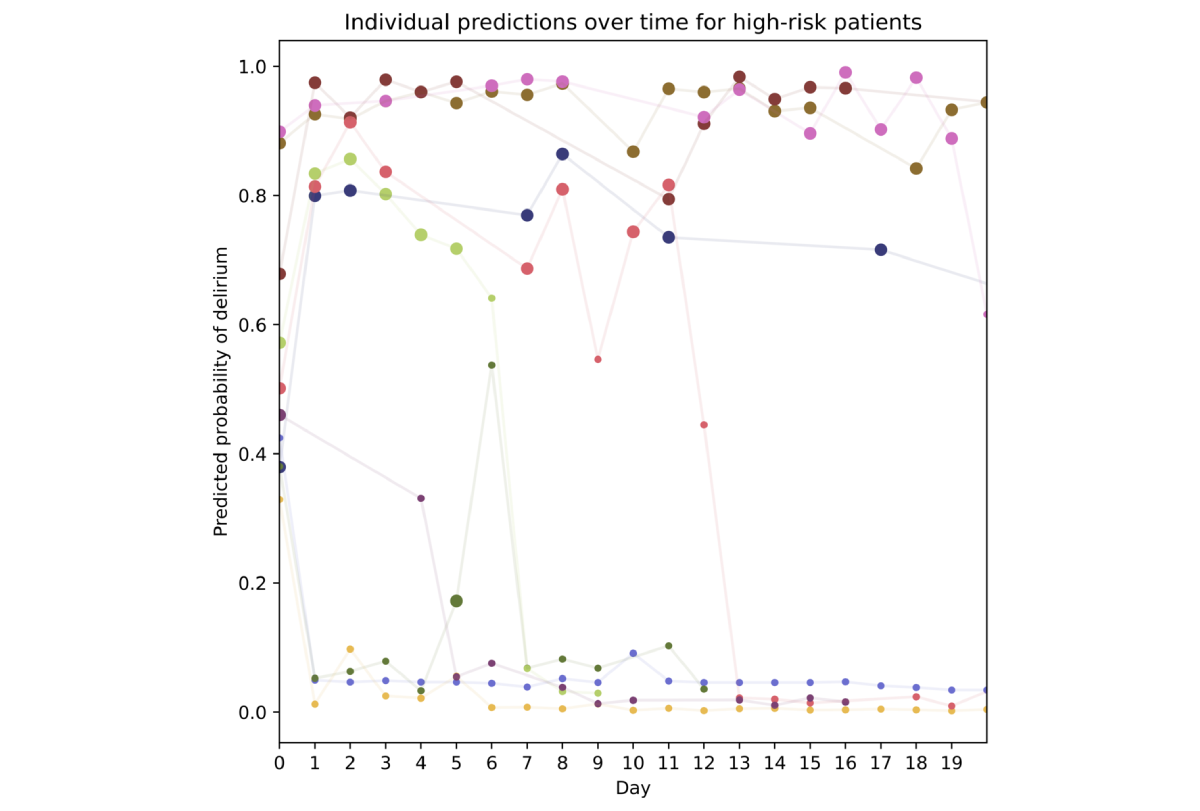
Predicted versus actual delirium incidence for ten patients with elevated (>30%) initial risk of delirium using predictions from the boosted tree model. Each patient is plotted in a different color, with a large dot reflecting at least one positive CAM screen that day and a small dot reflecting all negative CAM screens that day. The vertical axis shows the predicted risk for delirium that day (ie, for a perfect predictor, all large dots would be at the top of the plot and all small dots at the bottom). The horizontal axis shows the time (in days) from the first delirium screen, with day 0 being the day of the first delirium screen. CAM: Confusion Assessment Method.

To explore the dynamics of delirium predictions in individual patients over time, we examine the predicted risk and actual occurrence of positive CAM screens over time for a small number of patients with an elevated initial risk of delirium using delirium predictions from the boosted tree model (Figure 5). Of note, the prediction for the next day was strongly correlated with the delirium status of the previous day (as expected), but the first day without delirium usually had a lower predicted risk of delirium than the last day with delirium, suggesting that the model had identified changes correlated with resolution of the delirium.

## Discussion

### Principal Results

We have described the development of a prediction model that can provide automated daily predictions of the risk of delirium for a general population of hospitalized patients. We found that a boosted tree model performed best for this dataset and was able to identify a group of high-risk patients even when limited to patients who did not have delirium or had no prior history of delirium. However, the random forest, multilayer perceptron, and logistic regression models, while less effective, still provided substantial predictive power. All of the models showed good calibration on the full dataset but showed poorer MCE when applied to subsets of the data where delirium was less common—patients who did not currently have delirium and patients with no prior history of delirium.

The relative performance of the various models may reflect the relative match between the flexibility of each of the types of models and the size of the dataset we used. Although the L1 regularization used for the logistic regression model allowed for some tuning of flexibility by adjusting how many features were used, the logistic model can only capture monotonic relationships. Both boosted tree models and random forest models can better capitalize on nonmonotonic relationships which likely underlies their better performance on this dataset. In contrast, it was difficult to prevent overfitting with the multilayer perceptron model; preventing this overfitting would likely require either a larger dataset or additional methods of regularization.

### Limitations

All of the models showed a decrement in performance when restricted to patients who did not currently have delirium and a further decrement when restricted without prior delirium. Except for the multilayer perceptron in the no prior delirium case; however, the AUROC of all of the models remained greater than 0.75, which many would consider the threshold for “good” performance of a clinical test [31]. The boosted tree model, in particular, maintained an AUROC of 0.80, which compares very favorably to commonly used diagnostic tests such as D-dimer levels in the setting of a suspected pulmonary embolism (with a reported AUROC of 0.71 [32]). The decrement of performance in these subgroups likely reflects the increased difficulty of the task—those patients with significant risk factors are likely to have had delirium on prior days or hospitalizations and most of those who remain are relatively unlikely to become delirious in the next 24 hours. Even within the patients with no prior delirium, however, the boosted tree model was able to identify high-risk patients who would likely warrant further evaluation and interventions—for example, from the precision-recall curves (Figure 2), we can see that even if one were to demand a 50% true-positive threshold for an intervention, the model would correctly identify over 10% of the patients who have never had delirium who would become delirious as candidates for that intervention.

An additional limitation is the potential selection bias of the training and evaluation dataset used by this study. During the time period used, CAM screening was used universally on some inpatient units in the study hospital (eg, orthopedics) but not others (eg, general surgery), and thus, the study population is more reflective of these units than of the hospital as a whole. Thus, the generalizability of these results to other institutions will depend on how similar their patient population is to these units, rather than to the patient population of the study hospital. In addition, the results reported reflect this population where serial screens are performed and prior delirium screens are often present, which may not be applicable in many settings. One would expect the model’s performance to be worse in this setting (similar to what is seen in the results reported for patients with no prior delirium).

As with many machine learning models, more complex models such as the boosted tree model may trade accuracy for interpretability, for example, for a clinician trying to understand why a particular patient is at risk for delirium. While this can be partially addressed by including the relative contributions of each input to the model to a given patient’s risk (eg, using Shapley values [29,30]) as part of the delirium risk report for each patient, this still can hide complex interactions between risk factors that may be important.

We have chosen to leave the predictions of the model in the form of a percentage risk, rather than simplifying the result to a binary prediction as is more familiar for many clinical tests. To the limits of calibration of the model, the predicted percentage of delirium can be interpreted as the positive predictive value of the test for that particular patient (and 100% minus the predicted percentage as the negative predictive value). For given interventions, it may make sense to set a threshold predicted risk based on a cost or benefit analysis of the intervention (thus reducing the prediction to a binary value with a single positive predictive value and negative predictive value for all patients); examinations of specific thresholds for specific interventions may be addressed in future work.

While we have included many potential input features in our prediction model, there are many additional features in the medical record that we have not attempted to use, such as flowsheet data, length of stay, and unstructured data such as clinical notes. In addition, we have not exhaustively explored the types of models available in the literature, including many regularization techniques (such as early stopping and L2 normalization). While future models incorporating these predictors and techniques may perform even better than the models described here, this work provides a lower bound for their performance.

### Comparison With Prior Work

Multiple prior prediction models for delirium have been developed for use in intensive care unit patients [11,12,33] and in hospitalized older patients [9]. In general, these models use a small number of predictors (4-11) identified using logistic regression, including such predictors as age, history of cognitive impairment, history of alcohol abuse, respiratory failure, blood urea nitrogen, mean arterial pressure, use of corticosteroids, admission category, admission urgency, and vision impairment. Our prediction goal (predicting a positive CAM screen within the next 24 h) is somewhat different than the existing models we are aware of, as it is aimed at the specific task of helping determine which hospitalized patients should be seen by a delirium service that day. With that caveat, the performance of our model appears to compare well with other models on similar tasks. Chua et al [13] provide a good review of similar models; reported AUROCs in this review range from 0.71 to 0.91 (compared to 0.92 for the boosted tree model we report). As delirium is an infrequent event, however, the AUPRC may provide a better estimate of the model’s performance as a screening tool. Comparing our model to the best-performing models in the review by Chua et al [13], only the model described by Corradi et al [34] (one of the two models with an AUROC of 0.91) reported an AUPRC, which was 0.60 (compared to 0.73 for the boosted tree model we report).

One of the challenges machine learning has faced in medicine is translating predictions into improvements in patient outcomes [35]. We plan to use this model to screen all of the patients in a 1000-bed hospital (which would be prohibitively labor-intensive to do by hand) and identify a set of high-risk patients to be visited by a delirium service. The members of this delirium service will then evaluate patients and provide recommendations to the team caring for the patient on how to reduce that patient’s risk of delirium. By focusing this additional clinical effort and possible interventions on the patients who would most likely benefit from them, we hope to use this tool to improve care at a lower cost per patient than providing the same interventions to every patient (including those at much lower risk of delirium).

Because of this intended use, we have made trade-offs that may limit the use of this model in other contexts. For example, our focus was on maximizing the ability of the model to identify high-risk hospitalized patients rather than on identifying the causal mechanism for a given patient’s delirium. Thus, for example, an arterial blood gas showing mild hyperoxia might be used for prediction by the model because it is correlated with intubation, sedation, and critical illness rather than because it is directly increasing the patient’s risk for delirium, and blindly attempting to correct this laboratory value may not decrease the patient’s risk of delirium. In addition, some features, such as administering an antipsychotic medication, may happen to treat an agitated delirium that has not been documented in the medical record; while this may still be quite useful for a delirium service, it may be less useful for a responding clinician who is treating the agitation who likely already knows he or she is treating a symptom of delirium. This is an example of a “shortcut feature” as described by Bellamy et al [36], where a causal connection is present in the training data (eg, the use of an antipsychotic for a patient the clinician has already decided has delirium) may not be present in a desired use-case of the model (eg, a clinician trying to decide whether a patient is at risk of delirium). Thus, while the model works well for finding high-risk patients, interpreting these risk factors and identifying appropriate interventions will still require clinical expertise.

Although our model can be applied in its current form, there are limitations that a user will need to be mindful of. For some machine learning models, such as logistic regression, it can be relatively easy to understand a prediction from the model in terms of the individual features contributing to the prediction. For many others, however, including boosted tree models such as our best-performing model, the nonlinear interaction of many features can make it difficult to understand why a given patient was assigned a high or low-risk score. Providing interpretability for these more complex, nonlinear models is an active area of research in machine learning, and while tools such as SHAP can provide some insight into a model, for some uses, a less accurate but more interpretable model (such as logistic regression) may be preferred.

Another limitation of our model is that many of the risk factors such as sleep disruption may only be documented in clinical notes and not in the structured data we have used, and thus high-risk patients may be missed by the model. We hope to address this in future work by integrating natural language processing techniques into the model.

The dataset used for the development and validation of the model is another potential source of bias in this study. Because the patients from this study are only those patients from a single academic medical center who received delirium screens, they may not be reflective of patients in other settings, and potentially not even representative of patients at the same institution who did not receive delirium screens. Although this could raise concerns that this would bias our dataset toward patients with delirium, this does not appear to have been the case. Only 4583 of the 23,006 patients (Table 1), or about 20%, of the patients in our dataset had one or more positive CAM screens. This is consistent with the 23% (95% CI 19%-26%) incidence of delirium reported in the meta-analysis of estimates of delirium occurrence reported by Gibb et al [37]. Although this is reassuring, future studies will be needed to provide external validation of the model at other institutions and on other patient populations.

### Conclusions

In this paper, we have described a method for predicting delirium in hospitalized patients given the information already present in the electronic medical record. A large dataset of over 23,000 patients allowed us to consider a larger number of candidate features while still allowing for rigorous validation with a blinded test dataset. The resulting model provides both good accuracy and good calibration and can be run in an automated fashion on data in the electronic patient record without requiring additional human effort. We believe this model can be of use in guiding clinicians and researchers in focusing on patients at greatest risk of delirium in hopes of mitigating the morbidity and mortality associated with this disease.

## Appendix

Multimedia Appendix 1 Details of the model development.

Multimedia Appendix 2 Details of the analysis.

Multimedia Appendix 3 Flowchart showing assignment of delirium status.

Multimedia Appendix 4 Missing fraction of top 100 features from boosted tree model.

## Abbreviations

AUPRC: area under the precision-recall curve
AUROC: area under the receiver operator characteristic curve
CAM: Confusion Assessment Method
ECE: expected calibration error
MCE: maximum calibration error
SHAP: Shapley Additive Explanations
TRIPOD: Transparent Reporting of a multivariable prediction model for Individual Prognosis Or Diagnosis
