# Supplementary material for: Daily Automated Prediction of Delirium Risk in Hospitalized Patients: Model Development and Validation
Source: JMIR Med Inform. 2025 Apr 18;13:e60442. doi: 10.2196/60442 (PMC12048784; doi:10.2196/60442)
Supplement: Multimedia Appendix 1 [file medinform_v13i1e60442_app1.docx]

#

## Patient features provided to the model

Age, gender, and all recorded vital signs, laboratory values, medications, and prior CAM assessments were used as input data to the model. We used all historical data available; in some cases (e.g. laboratory values) historical data was present as early as 2008, while in others (e.g. CAM screens) data was only available from 2016 and later. To avoid limiting the algorithm to a set of hand-selected features, we included all medications and laboratory vales at this stage, including rare ones (e.g. anti-onion IgE), resulting in 4863 unique laboratory tests and 5289 unique medication formulations. Due to the number of unique medications and formulations, we did not attempt to combine different formulations of the same medication into a single value.

## Data collection and preprocessing of data

All features were next converted from the raw text in our EMR into real (floating point) values. The raw text at times included comments or long-form text for laboratory reports, and an automated scoring system needed to be able to accept these inputs without human intervention. While this approach can yield noisy data in some cases, we reasoned that noisy data is tolerable for prediction purposes when it contains sufficient signal to improve the model accuracy. We thus adopted the simple but robust approach of using the first numerical value found in the text of the laboratory or vitals entry. If no numerical value was present the value was set to 1 if the word “positive” was found in the text, 0 if the word “negative” was found in the text, and otherwise discarded. If two values were found separated by a “/” (e.g. blood pressure), the second value was included as a second feature (with a "_slashvalue" suffix added to the name). This process resulted in a list of measurement containing tuples consisting of a patient MRN, timestamp, the feature measured, and a value.

Snapshots were generated summarizing the data available at 5AM each morning (i.e. before typical AM rounds) of every patient who went on to receive at least one CAM screen in the next 24 hours, resulting in 150281 snapshots in the training dataset and 37612 in the holdout dataset. Each snapshot consisted of the patient age (in days), gender (1 if male, 0 otherwise), and a series of summary statistics calculated for each of the measurements from the previous step (labs, medications, and vitals). For laboratory values and vitals, these consisted of the all-time minimum, all-time maximum, most recent, 24-hour mean, 24-hour standard deviation, 24-hour maximum, 24-hour minimum, and difference between the 24-hour maximum and minimum; all 24-hour statistics were calculated over the 24 hours prior to and including the snapshot time. When a statistic could not be calculated (e.g. a laboratory value had not been checked in the interval of interest, a 24-hour standard deviation could not be calculated because a value had been measured only once over the previous 24 hours, etc) it was marked as “missing”. Because the absence of an entry for a medication indicates a known (zero) dose rather than a missing measurement, a distinct set of statistics was used for medications, consisting of the all-time maximum dose, the latest dose, the total dose given over the past 24 hours, and the maximum dose in the past 24 hours. This resulted in a table with one row for each patient and for each day that CAM data was available for that patient, and one column for each summary statistic (60,090 columns).

For each snapshot an outcome measure was also generated, which was given a value of 1 if at least one of the CAM screens during the subsequent 24 hours was positive, and 0 if all of the CAM screens during that time were negative.

We next cleaned and normalized these values for use by the machine learning algorithms. Most machine learning algorithms are unable to handle missing values, and perform best when all values are of a similar order of magnitude. In addition, most of the features we identified in the previous stage are very sparse, and empirically we found that including these sparse features dramatically increased the computational work of the algorithms without improving their accuracy. We thus first used the training data for each model to identify features where over 95% of the values were missing or zero and removed these features from the data set. Note that this often involved keeping only some of the statistics for a given lab or medication, for example most (>55%) patients had a value for their most-recent glycosylated hemoglobin A1c, but very few (< 1/1000) had the multiple measurements within the past day required to calculate a 24-hour standard deviation statistic for this laboratory value.

We next used the training data to estimate the first, second, and third quartiles for each measure using the P2 algorithm [1]. Values that were more than 6 interquartile distances from the median were considered outliers and discarded (marked as missing). Because some values (e.g. total daily doses of uncommon medications) may have a value of 0 for more than 75% of the patients and yet are still of interest, when the third quartile for a feature was zero we would instead use the third quartile of the non-zero values of that feature when calculating interquartile intervals. The data was then normalized by subtracting the median and dividing by the interquartile range. Missing values were replaced with the normalized median value for that feature (i.e. 0).

The result of this preprocessing stage is a set (one per fold of cross validation) of new tables with one row per patient per day with CAM data available, and one column for each of ~2000 features (with small variations in which features are included for models trained on different folds of the data), with each feature having a median of 0, a maximum less than or equal to 6, a minimum greater than or equal to -6, and an interquartile range of approximately 1.

## Model training

The next stages depend on parameters “learned” from the data. Because fitting parameters to the same data they are tested on introduces a risk of over-fitting the data, 10-fold cross validation was used [2]. Briefly, patients were divided into 10 groups or “folds”, and the model was trained on data from 9 of the folds (the “training data set”) and then evaluated on the 10th fold (the “validation data set”). This process was repeated leaving out each fold in turn, generating a total of 10 models, each tested with data it had not been trained on. This approach reduces bias compared to fitting and testing on the same data set, and provides an estimate of sensitivity/variability (e.g. influence of sampling error on the model’s performance).

The XGBoost library [3] was used to fit boosted tree models [[4]} for the cleaned and normalized training data sets. For comparison, random forest models [5] and logistic regression models using L1 (i.e. LASSO) regularization [6] were also fit to the data using scikit learn [7]. In addition, a deep neural network model was developed using TensorFlow [8]. The final network had a 32-node RELU [9] input layer, two hidden layers of 16 and 8 RELU nodes respectively, and an output layer with a single sigmoidal node. All layers were fully connected, and a 50% dropout [10] was used between layers. For the logistic regression and random forest models, Platt scaling was used to improve calibration of the model.

All development was done using the Ubuntu 18.04 distribution of Gnu-Linux. Data processing and analysis was performed using the Python [11] and Julia [12] programming languages.

## Evaluation dataset and statistical analysis

After the set of models had been finalized and all training parameters fixed, a final model was trained using all the training data and evaluated on the 20% of the data that had been set aside as test data. No changes to the set of models or training parameters were performed after this unblinding of the test data (to avoid the risk of unintentional overfitting of the data by the experimenter). All results reported in this manuscript are from this test dataset; nevertheless, results on the training data set are very similar.

Confidence intervals were calculated in python using bootstrapping with 1000 rounds, each resampling the individual snapshots with replacement.

## Subgroup analysis

Because both active delirium (defined as the most recent CAM screen being positive) and a prior history of delirium (defined as one or more prior positive CAM screens) are both strong predictors of delirium, models were also evaluated on their ability to predict delirium for patients who were not currently delirious (defined as the most recent CAM screen being negative) and with patients that had no prior history of delirium (defined as never having had a positive CAM screen). To optimize the performance of the models for this task, they were retrained on the relevant subset of patients in the training data before being tested against patients in the test data. For clinical use the prior CAM screen results could be used to select which model to use; thus this optimization should not result in overestimating the potential performance of the model.

## Model performance metrics

### Model discrimination.

The receiver operator characteristic (ROC) curve was used to evaluate the predictive power of the prediction models. Because most predictive tests produce a value (a probability) rather than a binary result, setting different thresholds for how high this value needs to be to be considered “positive” produces different false positive (fraction of negative events predicted to be positive) and true positive rates (fraction of positive events predicted to be positive). For a given predictor we can plot all possible tradeoffs between true and false positive rates as a curve, the ROC curve. The ROC curve is used to compare classifiers, as it provides a metric independent of the relative frequency of positive and negative examples in the chosen dataset. The area under this curve (Area Under the ROC, or AUROC) is equal to the probability that the model will give an arbitrarily chosen positive example a higher score than an arbitrarily chosen negative example. An ideal model has an AUROC of 1 and an uninformative model an AUROC of 0.5. We also evaluate the predictive power of the models using precision-recall (PR) curves. When a phenomenon is less common, false positive predictions may make up a large fraction of the total positive predictions even if specificity is high. Because the ROC and AUROC are independent of the frequency of positive and negative examples in the dataset they do not capture this relative magnification of the importance of false positives in cases with rare events. If we instead plot the tradeoff between precision (also known as positive predictive value, which is the fraction of results predicted to be positive that are actually positive) as a function of recall (also known as the true positive rate, as in the ROC), we end up with a similar curve known as the precision-recall curve. This PR curve may provide a more informative measure of model performance in populations where the rates of positive and negative cases are similar to those in the test dataset. As with the ROC curve, it is common to compare the Area Under the PR Curve (the AUPRC) for different models to compare their predictive ability; an ideal model has an AUPRC of 1.

### Model calibration

We use calibration curves to evaluate the calibration of the models. In clinical practice, it is often useful to have a model provide a probability rather than just a “positive” or “negative” result; for example, one might rule out a diagnosis if the probability was 1%, perform follow-up tests if the probability was 49 or 51%, and initiate treatment if the probability was 99%. To evaluate how well the model's predicted probability of a positive result corresponds to the actual frequency of a positive result, we can group samples by deciles of predicted probability and plot the frequency of positive results in each decile. The resulting plot is known as a calibration curve, and for a perfectly calibrated model the frequency should match the predicted probability for each bin. Multiple metrics can be used to distill the calibration curve into a single value for comparing models; we use the expected calibration error (ECE) and maximum calibration error (MCE). The expected calibration error gives the average difference across all samples between the predicted probability and the actual probability of events in its prediction decile, and thus provides a good measure of the typical performance of the model. The Maximum Calibration Error, on the other hand, provides an estimate of worst-case error by reporting the largest difference between the average predicted probability for a prediction decile and the average frequency of positive cases within that same decile.

## Model Development References

1. Jain R, Chlamtac I. The P2 algorithm for dynamic calculation of quantiles and histograms without storing observations. Communications of the ACM 1985 Oct;28(10):1076–1085. doi: [10.1145/4372.4378](https://doi.org/10.1145/4372.4378)

2. Stone M. Cross-Validatory Choice and Assessment of Statistical Predictions. Journal of the Royal Statistical Society: Series B (Methodological) 1974;36(2):111–133. doi: [10.1111/j.2517-6161.1974.tb00994.x](https://doi.org/10.1111/j.2517-6161.1974.tb00994.x)

3. Chen T, Guestrin C. XGBoost: A Scalable Tree Boosting System. Proceedings of the 22nd ACM SIGKDD International Conference on Knowledge Discovery and Data Mining San Francisco, California, USA: Association for Computing Machinery; 2016. p. 785–794. doi: [10.1145/2939672.2939785](https://doi.org/10.1145/2939672.2939785)

4. Friedman JH. Greedy function approximation: A gradient boosting machine. The Annals of Statistics 2001 Oct;29(5):1189–1232. doi: [10.1214/aos/1013203451](https://doi.org/10.1214/aos/1013203451)

5. Breiman L. Random Forests. Machine Learning 2001 Oct;45(1):5–32. doi: [10.1023/A:1010933404324](https://doi.org/10.1023/A:1010933404324)

6. Tibshirani R. Regression Shrinkage and Selection Via the Lasso. Journal of the Royal Statistical Society: Series B (Methodological) 1996;58(1):267–288. doi: [10.1111/j.2517-6161.1996.tb02080.x](https://doi.org/10.1111/j.2517-6161.1996.tb02080.x)

7. Pedregosa F, Varoquaux G, Gramfort A, Michel V, Thirion B, Grisel O, Blondel M, Prettenhofer P, Weiss R, Dubourg V, Vanderplas J, Passos A, Cournapeau D, Brucher M, Perrot M, Duchesnay É. Scikit-learn: Machine Learning in Python. The Journal of Machine Learning Research 2011 Nov;12(null):2825–2830. doi: [10.5555/1953048.2078195](https://doi.org/10.5555/1953048.2078195)

8. Abadi M, Barham P, Chen J, Chen Z, Davis A, Dean J, Devin M, Ghemawat S, Irving G, Isard M, Kudlur M, Levenberg J, Monga R, Moore S, Murray DG, Steiner B, Tucker P, Vasudevan V, Warden P, Wicke M, Yu Y, Zheng X. TensorFlow: A System for Large-scale Machine Learning. Proceedings of the 12th USENIX Conference on Operating Systems Design and Implementation Berkeley, CA, USA: USENIX Association; 2016. p. 265–283. doi: [10.5555/3026877.3026899](https://doi.org/10.5555/3026877.3026899)

9. Krizhevsky A, Sutskever I, Hinton GE. ImageNet Classification with Deep Convolutional Neural Networks. Commun ACM 2012;60:84–90. doi: [10.1145/3065386](https://doi.org/10.1145/3065386)

10. Srivastava N, Hinton G, Krizhevsky A, Sutskever I, Salakhutdinov R. Dropout: A Simple Way to Prevent Neural Networks from Overfitting. Journal of Machine Learning Research 2014 Jun;15:1929–1958. doi: [10.5555/2627435.2670313](https://doi.org/10.5555/2627435.2670313)

11. Rossum G van, Boer J de. Linking a stub generator (AIL) to a prototyping language (Python). Proceedings of the Spring 1991 EurOpen Conference, Troms, Norway 1991. p. 229–247.

12. Bezanson J, Edelman A, Karpinski S, Shah V. Julia: A Fresh Approach to Numerical Computing. SIAM Review 2017 Jan;59(1):65–98. doi: [10.1137/141000671](https://doi.org/10.1137/141000671)
