# Supplementary material for: Daily Automated Prediction of Delirium Risk in Hospitalized Patients: Model Development and Validation
Source: JMIR Med Inform. 2025 Apr 18;13:e60442. doi: 10.2196/60442 (PMC12048784; doi:10.2196/60442)
Supplement: Multimedia Appendix 2 [file medinform_v13i1e60442_app2.docx]

#

## Evaluation dataset

After the set of models had been finalized and all training parameters fixed, a final model was trained using all the training data and evaluated on the 20% of the data that had been set aside as test data. No changes to the set of models or training parameters were performed after this unblinding of the test data (to avoid the risk of unintentional overfitting of the data by the experimenter). All results reported in this manuscript are from this test dataset; nevertheless, results on the training data set are very similar.

Confidence intervals were calculated in python using bootstrapping with 1000 rounds, each resampling the individual snapshots with replacement.

## Subgroup analysis

Because both active delirium (defined as the most recent CAM screen being positive) and a prior history of delirium (defined as one or more prior positive CAM screens) are both strong predictors of delirium, models were also evaluated on their ability to predict delirium for patients who were not currently delirious (defined as the most recent CAM screen being negative) and with patients that had no prior history of delirium (defined as never having had a positive CAM screen). To optimize the performance of the models for this task, they were retrained on the relevant subset of patients in the training data before being tested against patients in the test data. For clinical use the prior CAM screen results could be used to select which model to use; thus this optimization should not result in overestimating the potential performance of the model.

## Model performance metrics

### Model discrimination.

The receiver operator characteristic (ROC) curve was used to evaluate the predictive power of the prediction models. Because most predictive tests produce a value (a probability) rather than a binary result, setting different thresholds for how high this value needs to be to be considered “positive” produces different false positive (fraction of negative events predicted to be positive) and true positive rates (fraction of positive events predicted to be positive). For a given predictor we can plot all possible tradeoffs between true and false positive rates as a curve, the ROC curve. The ROC curve is used to compare classifiers, as it provides a metric independent of the relative frequency of positive and negative examples in the chosen dataset. The area under this curve (Area Under the ROC, or AUROC) is equal to the probability that the model will give an arbitrarily chosen positive example a higher score than an arbitrarily chosen negative example. An ideal model has an AUROC of 1 and an uninformative model an AUROC of 0.5. We also evaluate the predictive power of the models using precision-recall (PR) curves. When a phenomenon is less common, false positive predictions may make up a large fraction of the total positive predictions even if specificity is high. Because the ROC and AUROC are independent of the frequency of positive and negative examples in the dataset they do not capture this relative magnification of the importance of false positives in cases with rare events. If we instead plot the tradeoff between precision (also known as positive predictive value, which is the fraction of results predicted to be positive that are actually positive) as a function of recall (also known as the true positive rate, as in the ROC), we end up with a similar curve known as the precision-recall curve. This PR curve may provide a more informative measure of model performance in populations where the rates of positive and negative cases are similar to those in the test dataset. As with the ROC curve, it is common to compare the Area Under the PR Curve (the AUPRC) for different models to compare their predictive ability; an ideal model has an AUPRC of 1.

### Model calibration

We use calibration curves to evaluate the calibration of the models. In clinical practice, it is often useful to have a model provide a probability rather than just a “positive” or “negative” result; for example, one might rule out a diagnosis if the probability was 1%, perform follow-up tests if the probability was 49 or 51%, and initiate treatment if the probability was 99%. To evaluate how well the model's predicted probability of a positive result corresponds to the actual frequency of a positive result, we can group samples by deciles of predicted probability and plot the frequency of positive results in each decile. The resulting plot is known as a calibration curve, and for a perfectly calibrated model the frequency should match the predicted probability for each bin. Multiple metrics can be used to distill the calibration curve into a single value for comparing models; we use the expected calibration error (ECE) and maximum calibration error (MCE). The expected calibration error gives the average difference across all samples between the predicted probability and the actual probability of events in its prediction decile, and thus provides a good measure of the typical performance of the model. The Maximum Calibration Error, on the other hand, provides an estimate of worst-case error by reporting the largest difference between the average predicted probability for a prediction decile and the average frequency of positive cases within that same decile.
