# Supplementary material for: Daily Automated Prediction of Delirium Risk in Hospitalized Patients: Model Development and Validation
Source: JMIR Med Inform. 2025 Apr 18;13:e60442. doi: 10.2196/60442 (PMC12048784; doi:10.2196/60442)
Supplement: Multimedia Appendix 3 [file medinform_v13i1e60442_app3.docx]

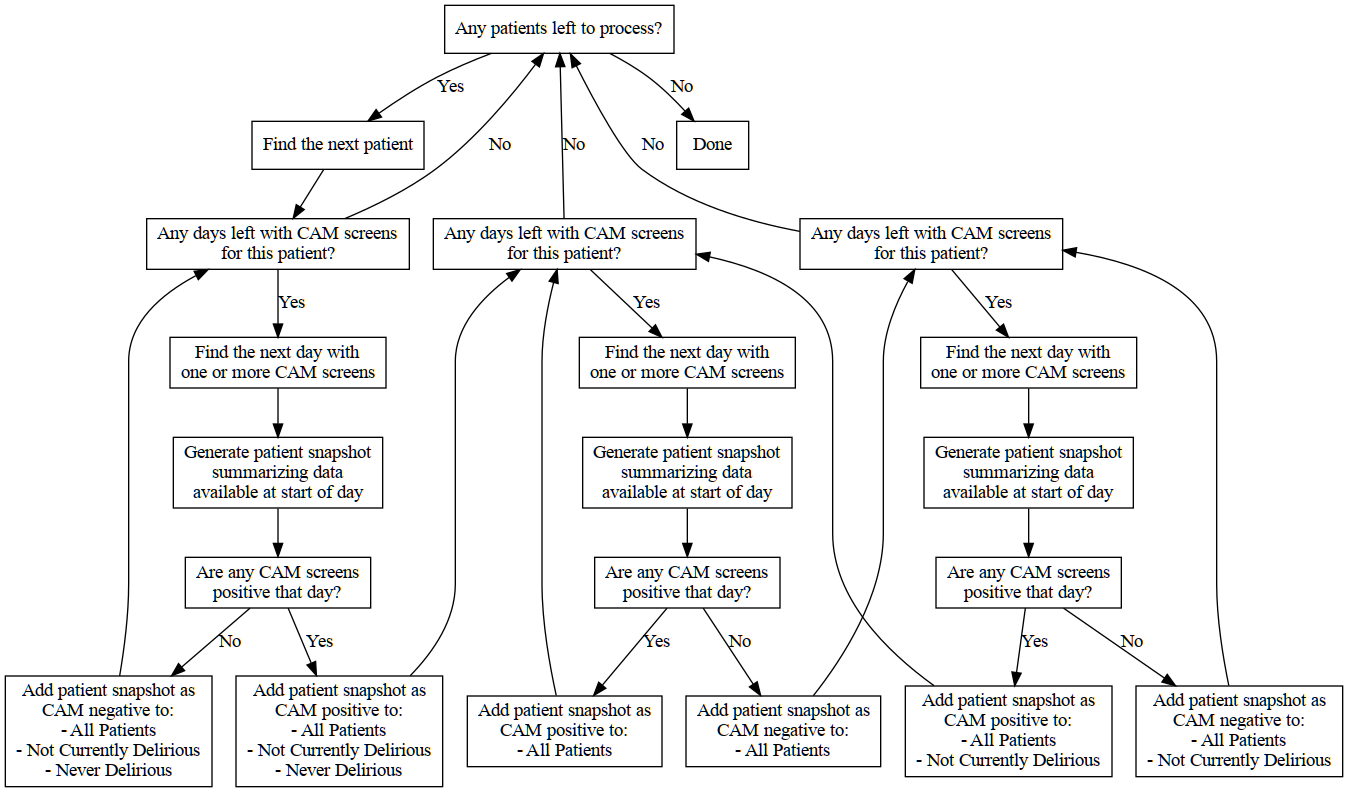

**Figure S1:** Conceptual flowchart for patient pathways. The patients are looped through in order. For each patient, the days with delirium screens are considered in chronological order. A patient is initially considered “never delirious (left path through flowchart), until a positive delirium screen is encountered. They are then considered delirious (middle path) until a day with all negative screens is encountered. They are then considered”previously but not currently delirious” (right path) until a positive delirium screen is encountered, at which point they transition back to the middle “delirious” pathway. Note that each patient may contribute snapshots in each category. The “Not delirious” category is the union of “Never delirious” and “previously but not currently delirious.”
