# Supplementary material for: Daily Automated Prediction of Delirium Risk in Hospitalized Patients: Model Development and Validation
Source: JMIR Med Inform. 2025 Apr 18;13:e60442. doi: 10.2196/60442 (PMC12048784; doi:10.2196/60442)
Supplement: Multimedia Appendix 4 [file medinform_v13i1e60442_app4.docx]

#

| Feature | Fraction Missing |
| --- | --- |
| CAM_alltimelatest | 0.13 |
| CAM_alltimemax | 0.13 |
| CAM_maxmindiff | 0.28 |
| CHLORHEXIDINE GLUCONATE 0.12 % MOUTHWASH_maxdose | 0 |
| CAM_mean | 0.28 |
| PO2_maxmindiff | 0.9 |
| CAM_min | 0.28 |
| VASOPRESSIN INFUSION IN NS 1 UNIT/ML (50 ML) CENTRAL_alltimelatest | 0 |
| HALOPERIDOL LACTATE 5 MG/ML INJECTION SOLUTION_alltimelatest | 0 |
| FIO2_alltimemax | 0.73 |
| ACETAMINOPHEN_alltimemin | 0.79 |
| CISATRACURIUM 2 MG/ML INTRAVENOUS SOLUTION_alltimemax | 0 |
| CAM_std | 0.57 |
| EGFR_mean | 0.4 |
| URINE OXYCODONE_alltimemin | 0.88 |
| ETHANOL_alltimelatest | 0.78 |
| HALOPERIDOL LACTATE 5 MG/ML INJECTION SOLUTION_alltimemax | 0 |
| RIFAXIMIN 550 MG TABLET_alltimemax | 0 |
| CSF NEUTS_alltimemin | 0.94 |
| PCO2_maxmindiff | 0.9 |
| SODIUM CHLORIDE 0.9 % INTRAVENOUS SOLUTION_maxdose | 0 |
| OMEPRAZOLE 20 MG CAPSULE_DELAYED RELEASE_totaldose | 0 |
| PREDNISONE 20 MG TABLET_alltimemax | 0 |
| CHLORHEXIDINE GLUCONATE 0.12 % MOUTHWASH_totaldose | 0 |
| PO2_max | 0.9 |
| HBV SURFACE AB QUANT_alltimelatest | 0.82 |
| SODIUM CHLORIDE 0.9 % INTRAVENOUS SOLUTION_totaldose | 0 |
| PH_maxmindiff | 0.83 |
| NRBC_alltimelatest | 0.1 |
| URINE CANNABINOIDS_alltimemax | 0.73 |
| CLONIDINE HCL 0.1 MG TABLET_alltimemax | 0 |
| QUETIAPINE 25 MG TABLET_alltimemax | 0 |
| LOSARTAN 25 MG TABLET_alltimelatest | 0 |
| FIO2_min | 0.94 |
| TOTAL VOLUME_alltimemax | 0.92 |
| CEFEPIME IVPB 2 G (50 ML) MBP_totaldose | 0 |
| PLASMA CREATININE_alltimelatest | 0.68 |
| ASPIRIN 81 MG TABLET_DELAYED RELEASE_totaldose | 0 |
| DOCUSATE SODIUM 50 MG/5 ML ORAL LIQUID_totaldose | 0 |
| QUETIAPINE 25 MG TABLET_alltimelatest | 0 |
| ONDANSETRON HCL (PF) 4 MG/2 ML INJECTION SOLUTION_alltimemax | 0 |
| BASE EXCESS_ UNSPECIFIED_alltimelatest | 0.91 |
| AMINO ACIDS-PROTEIN HYDROLYSATE 15 GRAM-60 KCAL/30 ML ORAL LIQUID PACK_totaldose | 0 |
| OLANZAPINE 5 MG DISINTEGRATING TABLET_alltimemax | 0 |
| DEXAMETHASONE 4 MG/ML INJECTION SOLUTION_alltimemax | 0 |
| VASOPRESSIN INFUSION IN NS 1 UNIT/ML (50 ML) CENTRAL_alltimemax | 0 |
| OSMOLALITY_alltimemax | 0.81 |
| POTASSIUM CHLORIDE 20 MEQ/L IN D5-0.9 % SODIUM CHLORIDE INTRAVENOUS_alltimelatest | 0 |
| GADOTERATE MEGLUMINE 0.5 MMOL/ML INTRAVENOUS SYRINGE_alltimemax | 0 |
| QUETIAPINE 25 MG TABLET_maxdose | 0 |
| QUETIAPINE 25 MG TABLET_totaldose | 0 |
| PANTOPRAZOLE IVPB 40 MG (100 ML) MBP_alltimemax | 0 |
| MORPHINE 2 MG/ML INTRAVENOUS CARTRIDGE_alltimemax | 0 |
| IOPAMIDOL 76 % INTRAVENOUS SOLUTION_alltimemax | 0 |
| SPUTUM_alltimemin | 0.94 |
| PLASMA AMMONIA_alltimelatest | 0.77 |
| LEVETIRACETAM 500 MG TABLET_alltimemax | 0 |
| ALBUMIN_ HUMAN 5 % INTRAVENOUS SOLUTION_alltimelatest | 0 |
| LACTULOSE 20 GRAM/30 ML ORAL SOLUTION_totaldose | 0 |
| CSF NEUTS_alltimemax | 0.94 |
| Age | 0 |
| FLUID CHOLESTEROL_alltimemax | 0.95 |
| BASE EXCESS_alltimemax | 0.89 |
| PH_min | 0.83 |
| PLASMA ANION GAP_alltimelatest | 0.68 |
| POTASSIUM CHLORIDE ER 20 MEQ TABLET_EXTENDED RELEASE(PART/CRYST)_alltimemax | 0 |
| INFLUENZA B AG_alltimemax | 0.85 |
| LEVOTHYROXINE 100 MCG TABLET_alltimemax | 0 |
| ABSOLUTE NRBC_std | 0.84 |
| ACETAMINOPHEN 650 MG RECTAL SUPPOSITORY_alltimelatest | 0 |
| PROMYELO_alltimemax | 0.9 |
| FENTANYL (PF) 50 MCG/ML INJECTION SOLUTION_alltimelatest | 0 |
| ALBUMIN_min | 0.75 |
| DIRECT BILIRUBIN_alltimemax | 0.18 |
| CEFAZOLIN IVPB 1 G (50 ML) MBP_alltimemax | 0 |
| SPECIFIC GRAVITY_min | 0.91 |
| SUPERSTAT PT_alltimemax | 0.86 |
| FENTANYL (PF) 50 MCG/ML INJECTION SOLUTION_alltimemax | 0 |
| FLUID MONOS_alltimemax | 0.82 |
| HIGH DENSITY LIPOPROTEIN_alltimelatest | 0.79 |
| PLASMA AMMONIA_alltimemax | 0.77 |
| ABSOLUTE NRBC_alltimelatest | 0.1 |
| URINE OSMOLALITY_alltimemax | 0.77 |
| FIO2_alltimemin | 0.73 |
| FUROSEMIDE 20 MG TABLET_alltimelatest | 0 |
| NEUTS_alltimemin | 0.17 |
| VENOUS PH_alltimelatest | 0.9 |
| CHEMISTRY COMMENT_alltimelatest | 0.94 |
| FUROSEMIDE 10 MG/ML INJECTION SOLUTION_alltimelatest | 0 |
| PROTEIN_mean | 0.91 |
| ETHANOL_alltimemax | 0.78 |
| CSF EOS_alltimemin | 0.94 |
| FLUID EOS_alltimemin | 0.82 |
| CLONIDINE HCL 0.1 MG TABLET_alltimelatest | 0 |
| IBUPROFEN 600 MG TABLET_alltimemax | 0 |
| SODIUM_max | 0.39 |
| LYMPHS_min | 0.74 |
| BUN_alltimelatest | 0.13 |
| VANCOMYCIN 1 GRAM/200 ML IN DEXTROSE 5 % INTRAVENOUS PIGGYBACK_alltimemax | 0 |
| VITAMIN B12_alltimemax | 0.55 |
